# The Practitioner’s Hand Book of Treatment

**Published:** 1877-03

**Authors:** 


The following, from want of space, are merely mentioned, and recommended to favorable consideration.

THE PRACTITIONER'S HAND BOOK OF TREATMENT, OR THE PRINCIPLES OF THERAPEUTICS. By J. Milner Forthergill, M.D., M.R.C.P., Assistant Physician to the City of London Hospital for the diseases of the Chest, Victoria Park; Assistant Physician to the West London Hospital; 565 pages. Publishers, Henry C. Lea: Philadelphia. 1877.

